# A Dense RNN for Sequential Four-Chamber View Left Ventricle Wall Segmentation and Cardiac State Estimation

**DOI:** 10.3389/fbioe.2021.696227

**Published:** 2021-08-06

**Authors:** Yu Wang, Wanjun Zhang

**Affiliations:** ^1^Research Center for Physical Education Reform and Development, School of Physical Education, Henan University, Kaifeng, China; ^2^Henan Key Laboratory of Big Data Analysis and Processing, School of Computer and Information Engineering, Henan University, Kaifeng, China

**Keywords:** four-chamber view cardiac, recurrent neural network, image segmentation, left ventricle wall, cardiac state estimation

## Abstract

The segmentation of the left ventricle (LV) wall in four-chamber view cardiac sequential image is significant for cardiac disease diagnosis and cardiac mechanisms study; however, there is no successful reported work on sequential four-chambered view LV wall segmentation due to the complex four-chamber structure and diversity of wall motion. In this article, we propose a dense recurrent neural network (RNN) algorithm to achieve accurately LV wall segmentation in a four-chamber view MRI time sequence. In the cardiac sequential LV wall process, not only the sequential accuracy but also the accuracy of each image matters. Thus, we propose a dense RNN to provide compensation for the first long short-term memory (LSTM) cells. Two RNNs are combined in this work, the first one aims at providing information for the first image, and the second RNN generates segmentation result. In this way, the proposed dense RNN improves the accuracy of the first frame image. What is more is that, it improves the effectiveness of information flow between LSTM cells. Obtaining more competent information from the former cell, frame-wise segmentation accuracy is greatly improved. Based on the segmentation result, an algorithm is proposed to estimate cardiac state. This is the first time that deals with both cardiac time-sequential LV segmentation problems and, robustly, estimates cardiac state. Rather than segmenting each frame separately, utilizing cardiac sequence information is more stable. The proposed method ensures an Intersection over Union (IoU) of 92.13%, which outperforms other classical deep learning algorithms.

## 1. Introduction

The sequential segmentation of the left ventricle (LV) wall in a four-chamber view MRI image plays an important role in clinical disease diagnosis and physiological mechanism research works. Compared with a two-chamber view cardiac image, a four-chamber view image has distinctive advantages in cardiac assessment. For example, it is needed to compare the four chambers of cardiac in size and contractility to diagnose congenital heart disease (Copel et al., 1987); an LV sequence image can be used to evaluate the LV wall motion, which has been used to evaluate the risk of heart

failure (Konstam et al., 2011); Many studies have focused on the mechanisms of excitable tissue in cardiac based on the morphological feature of an LV wall (Constantino et al., 2010). Furthermore, a four-chamber view sequence is used to measure the relationship between left ventricular diastolic dysfunction and exercise intolerance in obese heart failure with preserving (Samuel et al., 2021), the cardiac state estimate is critical for the assessment of cardiac function and morphology. Thus, it is desired to propose a method to solve the sequential LV wall segmentation problem in a four-chamber view MRI image.

### 1.1. Challenges of LV Segmentation

**Figure 1:**
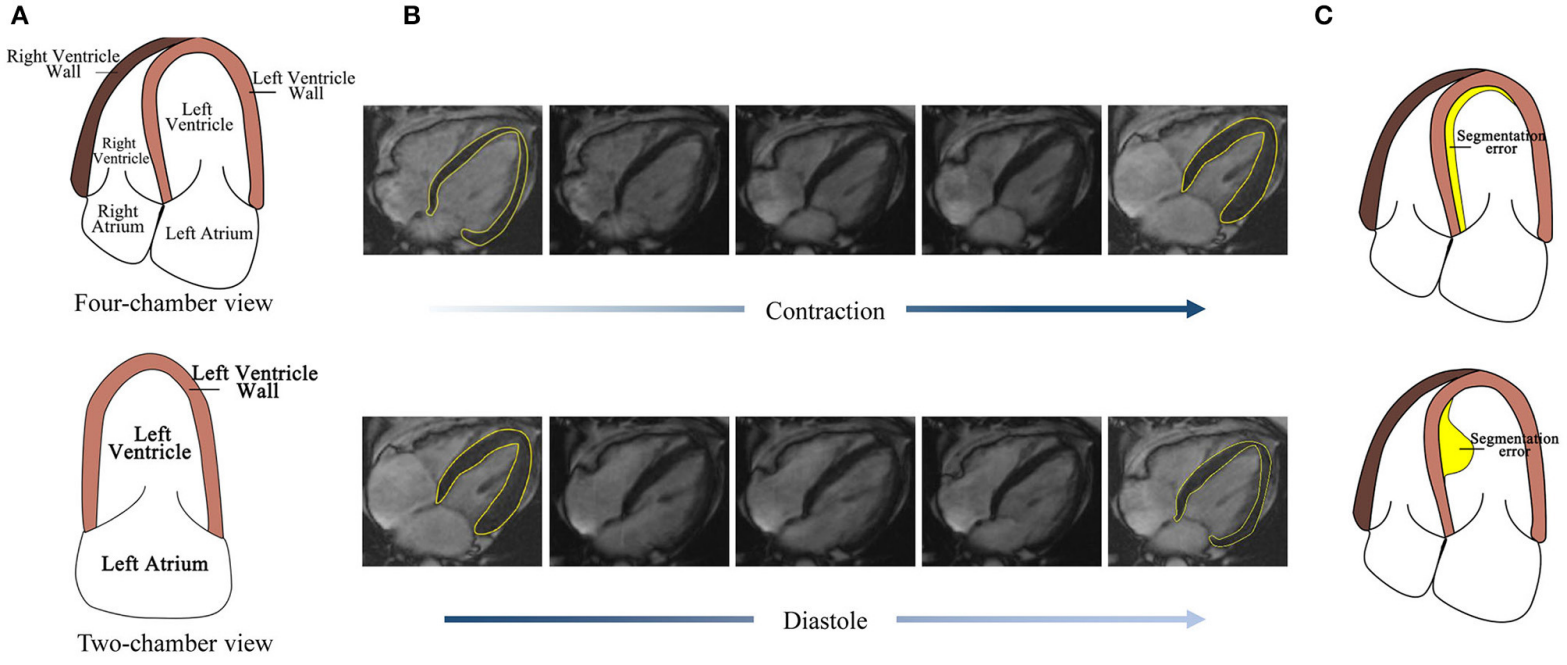
Challenges for four-chamber view MRI image segmentation. **(A)** The structure of the four-chamber view cardiac image is more complex than that of the two-chamber view. **(B)** The LV wall difference between frames caused by cardiac motion is small, thus high accuracy is needed. **(C)** Detection error with the same area but the different effect to its subsequent frames.

However, the automatic segmentation of LV wall in four-chamber view MRI images is still a challenge, as shown in Figure 1, (1) the complex structure of four-chamber view MRI image makes it hard to separate LV wall with other tissues. Figure 1A also shows that, compared with the two-chamber view cardiac image, the influence of the right ventricle (RV) and right atrium makes it harder to segment the LV wall. The similarity in intensity and structure feature with RV wall also increased the difficulty of LV wall segmentation. (2) High segmentation accuracy is desired in clinical for wall motion evaluation. Figure 1B shows LV wall changes at contraction state and diastole state. The difference between neighbor frames is small, which needs high accuracy on distinguishing contraction state and diastole state. (3) Segmentation error evaluation problem. Figure 1C shows that, the error area and IoU of the two error detection illustration examples are the same; however, in sequential image processing progress, their effect on clinical evaluation is different and may lead to misdiagnosis. Thus, a method is needed to be proposed to deal with time-sequential cardiac images and to achieve both frame-wise and sequence-wise accuracy.

### 1.2. Related Works

#### 1.2.1. Automated Cardiac Segmentation Methods

Unfortunately, there is no successful reported research work focusing on automatic sequential LV wall segmentation due to the challenges mentioned above. Most research studies focus on the assessment of LV sequence parameters (such as LV wall thickness, area, and so on) and the LV wall segmentation of a single frame. Recently, the deep learning method is widely used in medical image processing. An anatomically constrained neural network (ACNN) based cardiac image enhancement and segmentation method is proposed in Oktay et al. (2018). The method works well on MRI images. In most previous segmentation works (Tran, 2016; Oktay et al., 2018; Khened et al., 2019), each frame of the MRI sequence is processed independently. To achieve continuity of segmentation or quantification and take advantage of the sequence information, a correlation between different frame must be established. In the language generative deep learning method, sequence generate adversarial nets (SGAN) proposed in Yu et al. (2017) performs well in sequence processing and achieves a good result; however, this model can-not get a satisfactory result when it comes to sequence image segmentation.

#### 1.2.2. Deep Learning Architectures for Medical Image Segmentation

In the context of medical image segmentation method, convolutional neural networks (CNN)-based works have shown great potential in recent studies (Renard et al., 2020). In methods (Avendi et al., 2016; Ngo et al., 2017; Kasinathan et al., 2019; Ma and Yang, 2020), CNN and traditional methods (such as level set method and deformable models) are combined to achieve good segmentation accuracy. Fully convolutional networks (FCN) (Long et al., 2015) and U-net (Ronneberger et al., 2015) have achieved remarkable success in the image segmentation problem. Compared with original U-net, attention modules combined U-net (Dong et al., 2018; Schlemper et al., 2019), self-guided attention U-net (Ashish and Jose, 2021) has resulted in enhanced models for pixel-wise segmentation tasks. Based on U-net, a recurrent convolutional neural network (RCNN) is proposed in Alom et al. (2018). The feature accumulation in RCNN ensures better feature representation for segmentation tasks. In Peng et al. (2020), a deep snake method is proposed to segment image by controlling the movement of object boundaries. In Xie et al. (2020), a PolarMask method is proposed and contour of instance in a polar coordinate is predicted. These two methods are good at segment instance with less concave property.

#### Recurrent Neural Network

Recurrent Neural Network (RNN) (Zaremba et al., 2014) is specialized in processing sequential data. Long short term memory (LSTM) cell (Greff et al., 2015) or gated recurrent unit (GRU) Cho et al. (2014) are combined in RNN to transfer information. Promising results have been achieved by RNN or RNN variants in speech processing (Karita et al., 2019; Li et al., 2019), text generation (Pawade et al., 2018; Yang et al., 2019), classification (Premkumar et al., 2020), and image processing (Yao et al., 2019; Zhou et al., 2020). RNN has been used in Xue et al. (2017) regional wall thicknesses of LV.

Convolutional networks, such as FCN and U-net, focus on the problem of single-frame image processing. Compared with other neural networks, RNN is good at dealing with sequential image processing problems. The cardiac in MRI sequence contracts and diastole continuously. To make the best of information between frames, we choose RNN to achieve the LV wall segmentation goal.

### 1.3. Contributions

We proposed a dense RNN method to deal with the challenges and overcome the shortage of existing methods mentioned above. The details of highlights are as follows:

(1) It is the first time that an RNN successfully deals with a time-sequential cardiac segmentation problem. Existing methods focus on single frame segmentation or difference slice sequential segmentation. In this article, the time-sequential frames belong to one slice of cardiac, but with different cardiac state, which is able to observe cardiac structure change. Rather than segment a single image, segment sequence takes advantage of the relationship between frames, which makes it better to solve the complex structures and wall motion problems.
(2) A dense RNN is proposed to improve the effectiveness of the information transmitted between LSTM cells and achieve frame-wise accuracy. In RNN, the first frame acquires no hidden information, but its LSTM output is transmitted to the rest of the frames and plays a most important role. The proposed dense RNN contains two RNNs. The first RNN aims at providing dense hidden information for the first LSTM cell in the second RNN. The second RNN receives the hidden information and generates the segmentation result. This not only contributes to improving the accuracy of the first image but also makes the output of the first LSTM cell contain more useful information. It is the same with the subsequent cells. Thus, frame-wise accuracy is improved.
(3) Based on the LV wall segmentation result, an algorithm is proposed to determine the cardiac state. The algorithm uses mean cardiac change difference instead of the difference between frames, which is more robust. As the change of neighbor frames is relatively small, we first calculate the difference between two neighbor frames and then use the mean difference to determine the cardiac state.

This article is organized as follows: first, the background of cardiac LV segmentation and the existing method are introduced; second, the proposed method will be illustrated; third, the experimental result will be analyzed.

## 2. Method

The proposed network mainly contains two RNNs. In this section, RNN is first introduced. And then, the proposed dense RNN is described.

### 2.1. Recurrent Neural Network

Recurrent neural network is good at dealing with sequential information. It consists of several LSTM cells. Each unit receives a hidden layer from the former cell, together with the current input, the output layer is generated.

**Figure 2:**
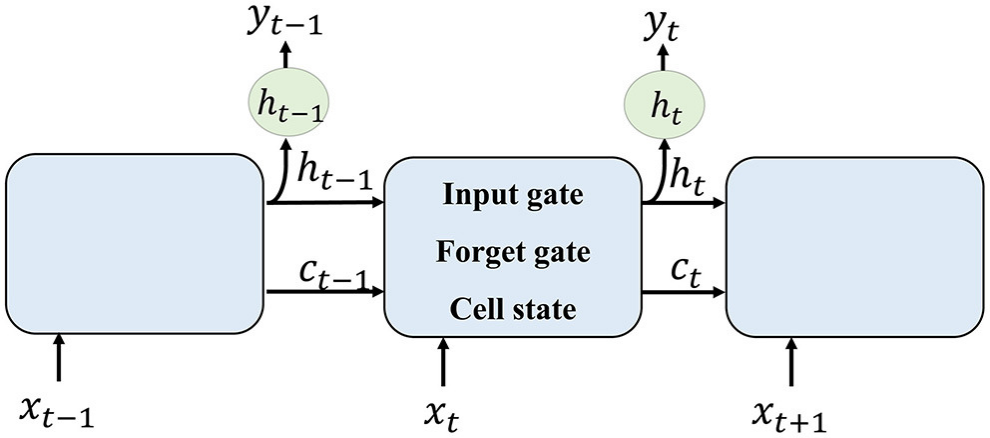
Recurrent neural network (RNN) method illustration.

The method of RNN is shown in Figure 2. Each LSTM cell receives information from the previous cell and transmits information to the next one. For an input image sequence *x*_1_, *x*_2_, …, *x*_*n*_, each *x*_*t*_ input into an LSTM cell. In general, an LSTM cell contains an input gate that generates *i*_*t*_, a forget gate that generates *f*_*t*_, and a memory vector *c*_*t*_. The relationship between these gates and the vector of LSTM can be denoted as follows:


(1) $$\begin{array}{r} \begin{array}{cc} f_{t} & =sigm\left(W_{f}\cdot \left[h_{t-1},x_{t}\right]+b_{f}\right)\\ i_{t} & =sigm\left(W_{i}\cdot \left[h_{t-1},x_{t}\right]+b_{i}\right)\\ \tilde{c_{t}} & =tanh\left(W_{o}\cdot \left[h_{t-1},x_{t}\right]+b_{o}\right) \end{array} \end{array}$$

where *h*_*t*−1_ is the hidden layer of the former cell and *b* is the bias factor.

Then, the memory cell of LSTM can be calculated as follows:


(2) $$\begin{array}{r} \begin{array}{c} c_{t}=f_{t}*c_{t-1}+i_{t}*\tilde{c_{t}} \end{array} \end{array}$$

where * is convolution transform. The output *o*_*t*_ and the hidden layer to the next LSTM are given by the following :


(3) $$\begin{array}{r} \begin{array}{cc} o_{t} & =sigm\left(W_{o}\cdot \left[h_{t-1},x_{t}\right]+b_{o}\right)\\ h_{t} & =o_{t}⊙tanh\left(c_{t}\right) \end{array} \end{array}$$

To formulate the segmentation result of one input *x*_*t*_, the current cell receives hidden layer *h*_*t*−1_ and memory vector *c*_*t*−1_ from former cell. The forget gate selects information that should be abandoned. Combining *h*_*t*−1_ and current input *x*_*t*_, the input gate selects new information that should be kept and generates a new cell state. The output of one cell is generated by the current input and formerly hidden layer.

### 2.2. Dense RNN

As illustrated in section RNN and shown in Figure 2, an LSTM cell *L*_*i*_ receives a hidden layer and memory cell from LSTM cell *L*_*i*−1_, together with input image *x*_*i*_, its segmentation image is generated. The first image in the sequence receives no information from the former cell. Thus, the accuracy of the first image is lower than the others. The hidden layer information and memory cell it flows to its following frames contain lower effective information.

**Figure 3:**
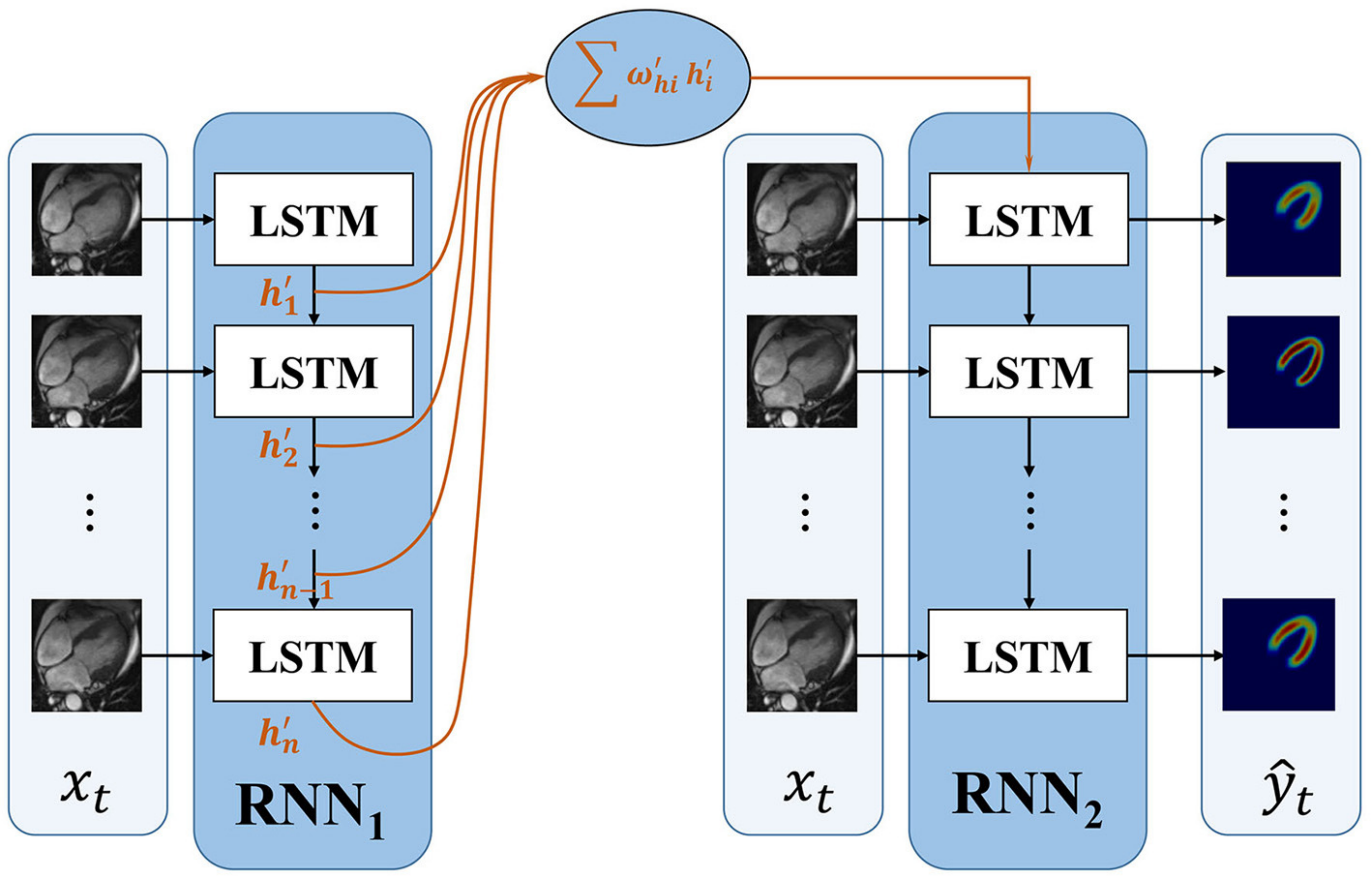
The framework of the proposed dense RNN.

To improve the accuracy of the first frame and make the information transmitted between frames more efficient, we propose an improved RNN. The proposed dense RNN is constituted by two RNNs. As shown in Figure 3, dense generator, main RNN (*RNN*_2_ in Figure 3) is used to generate segmentation results, while compensation RNN(*RNN*_1_ in Figure 3) provides compensation for the first LSTM cell in main RNN. With this compensation, the first frame acquires more information and makes the information flow in the main RNN more effective.

The first LSTM cell plays an important role in RNN. Its output information transmits to the subsequent LSTM cells; however, the first LSTM receives no hidden information, which means *h*_0_ = 0. The only input information it deals is *x*_1_. In this proposed dense RNN, the input hidden layer of the first LSTM cell in *RNN*_2_ can be denoted as follows:


(4) $$\begin{array}{r} h_{0}=\overset{n}{\underset{i=1}{\sum }}\omega_{hi}^{\prime }h_{n}^{\prime } \end{array}$$

where $h_{1}^{\prime },h_{2}^{\prime },\ldots ,h_{n}^{\prime }$ denote the output hidden layer of each LSTM in *RNN*_1_. The proposed dense RNN allows the network itself to choose the proper input hidden layer for the main RNN. The weight $\omega_{hi}^{\prime }$ in Equation 4 is trained by the network. With the weighted input hidden layer, the accuracy of the first frame is improved and the output of the first LSTM in the main RNN contains more useful information. In this way, the segmented accuracy of the subsequent frames is also improved.

The loss function is calculated by measuring the difference of ground truth and the output of the second RNN:


(5) $$\begin{array}{r} L=\overset{T}{\underset{t=1}{\sum }}\left(-y_{t}\log{ŷ}_{t}-\left(1-y_{t}\right)\log\left(1-{ŷ}_{t}\right)\right) \end{array}$$

where *y*_*t*_ is the ground truth of frame *t* and ŷ_*t*_ is the output of *RNN*_2_.

### 2.3. Cardiac State Estimation

The LV wall becomes thick during the contraction state and becomes thin during the diastole state. However, the change is not obvious for adjacent frames. In this article, we first calculate the difference between two adjacent frames as a reference. The difference of frame *k* and frame *k*+1 is defined by comparing the area of LV wall using Equation 6.


(6) $$d{\text{′}}_{k(k+1)}=\{\begin{array}{cc} 1, & if\;Area(f_{k})>Area(s_{k+1})\\ 0, & else \end{array}\;$$

where *Area*_(_*f*__*k*_)_ is the LV wall segmentation result of the area of the kth frame. Then, the cardiac state is estimated by the following equation:


(7) $$\begin{array}{r} s_{k\left(k+1\right)}=\frac{1}{N}\overset{k+N/2-1}{\underset{i=k-N/2}{\sum }}d_{i\left(i+1\right)}^{\prime } \end{array}$$

With the result of equation 7, *s*_*k*(*k*+1)_ <0.5 means that the cardiac frame is in a state of diastole, or else it is in a state contraction. By using Equation 7, the incorrect estimation by only two adjacent frames was reduced.

## 3. Experimental Results and Discussion

### 3.1. Dataset and Setting

The proposed method is tested using 137 groups of four-chamber view MRI cardiac images from 137 patients. Each group contains 18 continuous frames of the image, and every group contains frames of contraction and diastole cardiac states. The images are resized to 64 ×64. The ground truth of the LV wall is manually marked by doctors. We adopt 130 groups of images for training and the other 7 groups for testing. During training process, we use different frames as start frames to improve the robustness of the networks. The proposed network is implemented based on PyCharm and performed on NVIDIA Tesla P100.

### 3.2. Evaluation Metrics

The proposed method performs better than SeqGAN (Yu et al., 2017) and CNN method. The segmentation result is evaluated using the IoU factor. The IoU factor is obtained by $IoU=\frac{S\cap G}{S\cup G}$, where *S* is the segmentation result and *G* is the ground truth. ∩ and ∪ is the action of intersection and union.

### 3.3. Generation Performance

**Figure 4:**
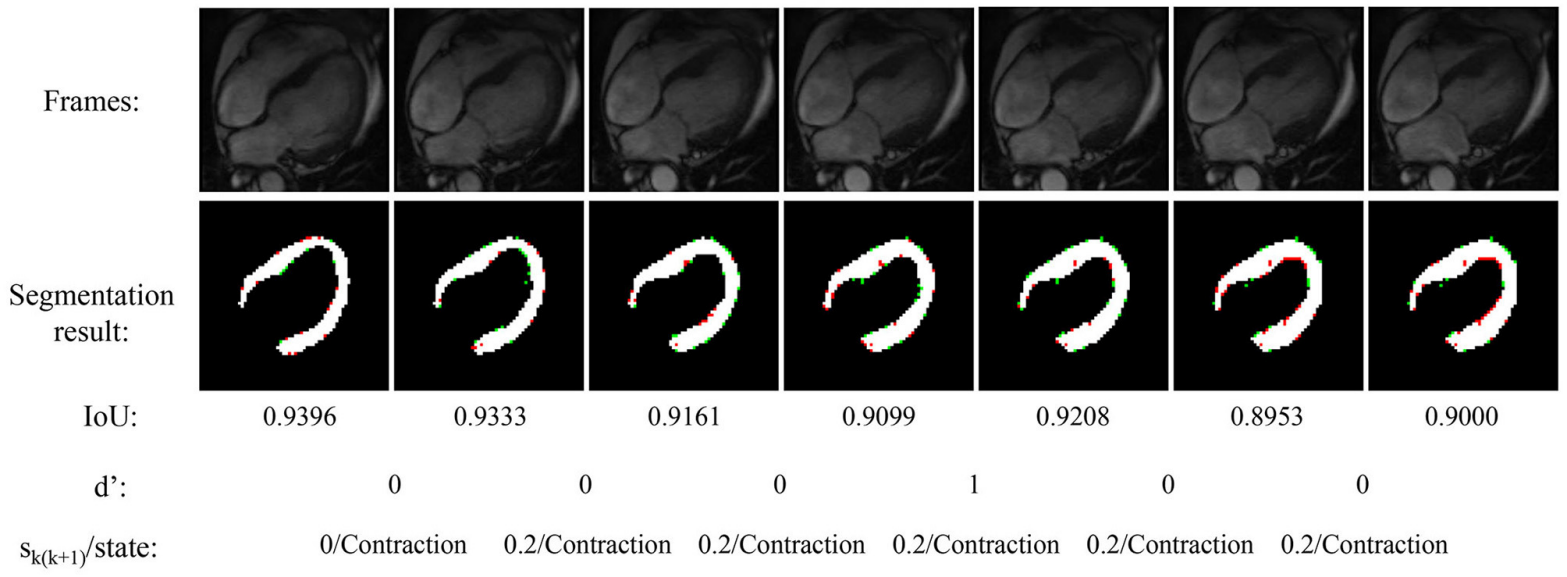
Experimental result of the proposed method during cardiac contraction. Red points in segmentation result: undetected LV pixels. Green points in segmentation result: error-detected pixels.

**Figure 5:**
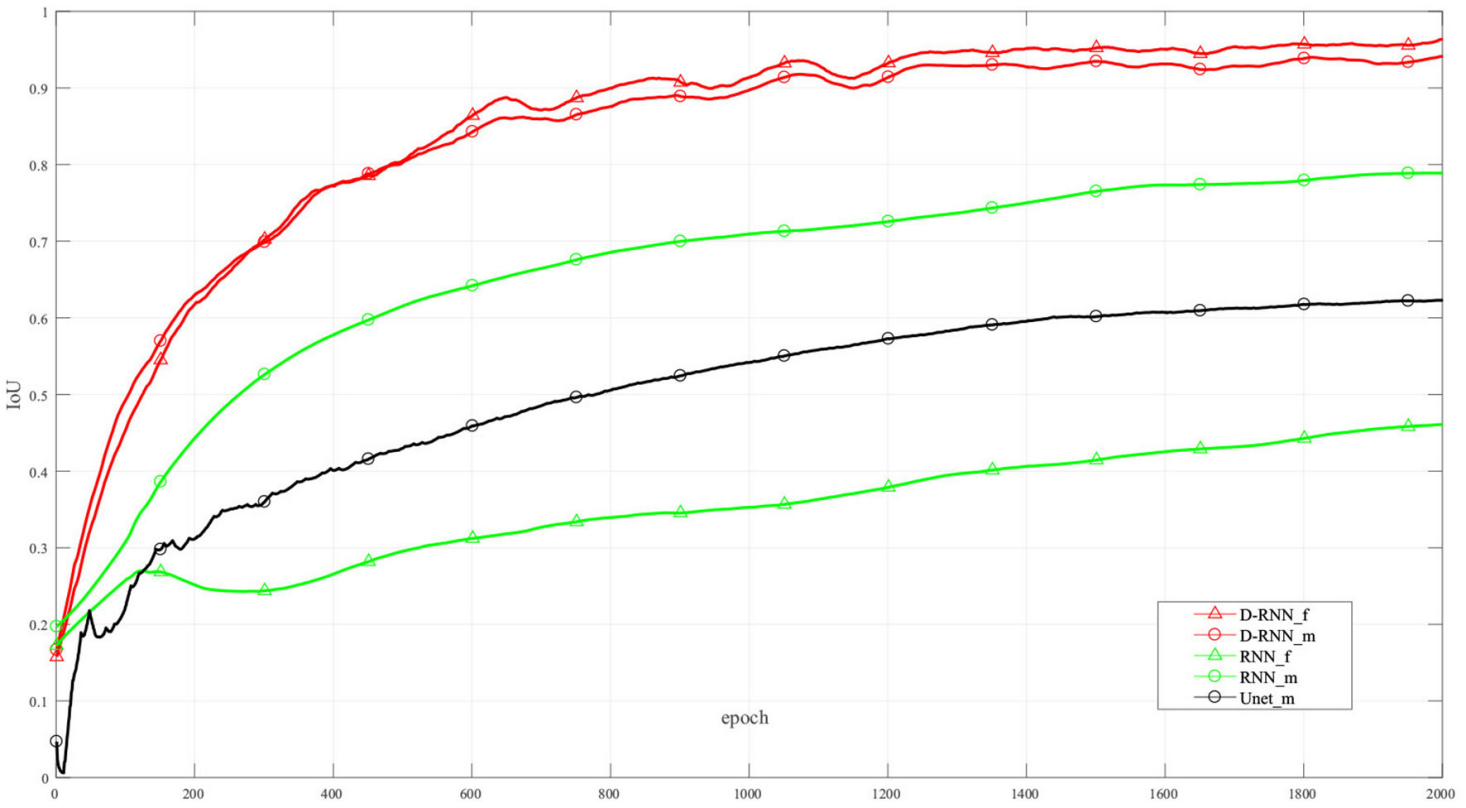
Segmentation result of the proposed method during cardiac diastole.

Figure 4 shows the experimental result of the proposed method. The frames in this sequence are during a contraction state, and the LV wall changes from thin to thick. The mean IoU of these frames is 91.64%. Though the changes between frames are little, the segmentation result reflex the change of LV wall thickness. Figure 5 shows the segmentation result during cardiac diastole. It can be seen from Figures 4 to 5 that the proposed method can obtain accurate diastole and contraction cardiac LV wall.

Figure 4 also illustrated the cardiac state method. All the frames are in a period of contraction. The difference of adjacent frames *d*′ incorrect estimate two frames as diastole with value 1. After the mean value calculated by equation 7, the incorrect estimate is corrected.

**Figure 6:**
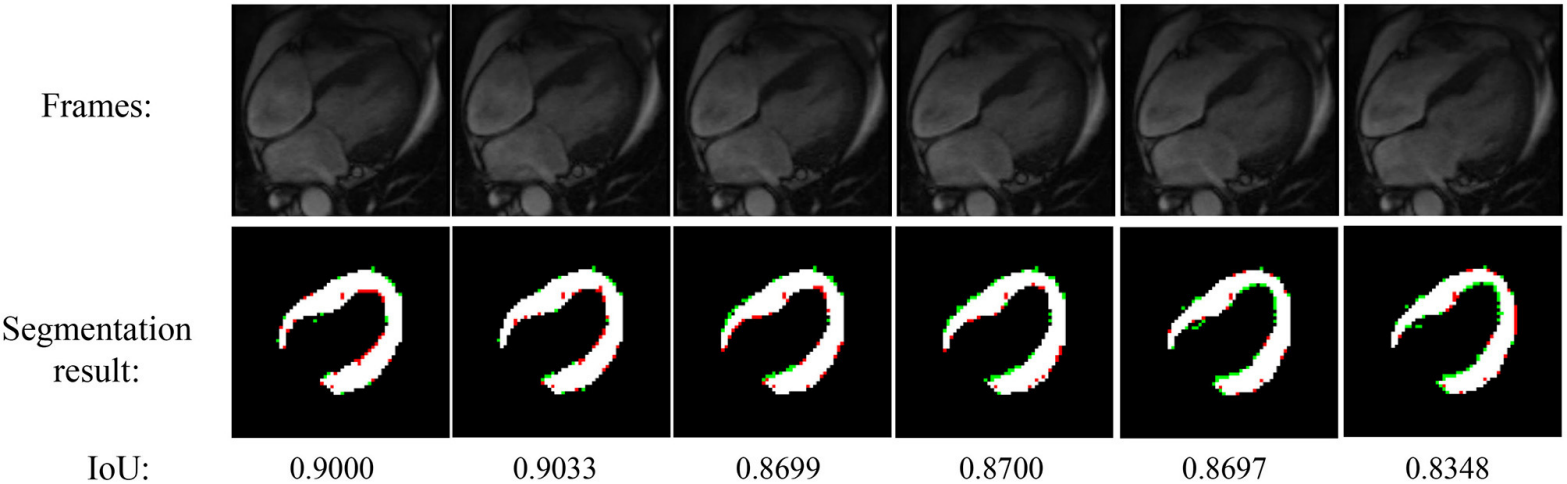
IoU at difference training interactions. _f: IoU of the first frame; _m: mean IoU of the sequence. For illustration propose, the line in this figure is filtered.

Figure 6 shows the IoU of the proposed method and other methods at different training interactions. The proposed method reaches an IoU of 92.13%, while the RNN method without dense net gets a result of 38.92%. We also use Unet (Ronneberger et al., 2015), a classic CNN deep learning method in image segmentation, to segment the LV wall. After 2000 times of interactions, the IoU reaches 61.75%. Compared with the other method, the proposed method highly improved the mean sequential segmentation accuracy.

The dense compensation for an RNN proposed in this method greatly improved the accuracy of the first frame in the sequence. Thus, the proposed method makes the hidden information flow in different LSTM cells more effectively, and the mean IoU is improved. It can be seen from Figure 6, the NoDense curve, that although the mean IoU by NoDense network is increasing with the increasing interaction, the IoU of the first frame remains unsatisfied. Each frame in the image sequence needed to be segmented with a promising result. The proposed dense RNN in DL-GAN works improves the mean accuracy and also ensures the accuracy of each frame. It can be seen from the DL-GAN curve in Figure 6 that the IoU factor of the first frame increases along with the mean IoU of the sequence. The mean accuracy is increased by 55.21% than the NoDense network.

## 4. Conclusion

In this article, a dense RNN method is proposed to segment the LV wall in a sequential four-chamber view MRI image. With the dense RNN, the segmentation accuracy of each frame in the sequence is guaranteed, and the accuracy of the first frame is greatly improved. The network reaches an IoU of 92.13%, which indicates the proposed method has prospects in cardiac disease diagnosis and cardiac mechanism analysis.

## Data Availability Statement

The original contributions presented in the study are included in the article/supplementary material, further inquiries can be directed to the corresponding author/s.

## Author Contributions

YW contributed to the conception of the study and data analysis. WZ performed the experiment and wrote the manuscript. Both authors contributed to the article and approved the submitted version.

## Conflict of Interest

The authors declare that the research was conducted in the absence of any commercial or financial relationships that could be construed as a potential conflict of interest.

## Publisher's Note

All claims expressed in this article are solely those of the authors and do not necessarily represent those of their affiliated organizations, or those of the publisher, the editors and the reviewers. Any product that may be evaluated in this article, or claim that may be made by its manufacturer, is not guaranteed or endorsed by the publisher.
