# Erratum: A Dense RNN for Sequential Four-Chamber View Left Ventricle Wall Segmentation and Cardiac State Estimation

**DOI:** 10.3389/fbioe.2021.811396

**Published:** 2021-11-25

**Affiliations:** Frontiers Media SA, Lausanne, Switzerland

**Keywords:** four-chamber view cardiac, recurrent neural network, image segmentation, left ventricle wall, cardiac state estimation

Due to a typesetting error, the reference for “Premkumar et al. (2020)” was incorrectly written as **Premkumar, K. A. R., Bharanikumar, R., and Palaniappan, A. (2020). Riboflow: Using deep learning to classify riboswitches with 99**. It should be “Premkumar, K. A. R., Bharanikumar R., and Palaniappan, A. (2020) Riboflow: Using Deep Learning to Classify Riboswitches With ∼99% Accuracy. *Front. Bioeng. Biotechnol*. 8:808. doi: 10.3389/fbioe.2020.00808”.

The publisher apologizes for this mistake. The original version of this article has been updated.
